# Drivers and Barriers for Implementation and International Transferability of Sustainable Pop-up Living Systems

**DOI:** 10.1007/s43615-021-00063-8

**Published:** 2021-06-21

**Authors:** Gaetano Bertino, Gloria Rose, Johannes Kisser

**Affiliations:** 1 Alchemia-Nova GmbH, Vienna, Austria; 2 Department of Water, Atmosphere and Environment, Institute of Sanitary Engineering and Water Pollution Control, University of Natural Resources and Life Sciences, Vienna, (BOKU) A-1190 Austria; 3 Institute of Technology Assessment of the Austrian Academy of Sciences, Vienna, Austria

**Keywords:** Pop-up environments, Temporary architecture, Pop-up housing, Sustainable construction, Building circularity

## Abstract

In the current urban system, characterised by a one-directional flow of resources from the rural environment to the cities, the construction sector plays a critical role in supporting the transition from a linear to a circular economy. In this framework, temporary pop-up environments act as an innovative and sustainable type of living system. These are structures conceived as temporary from the outset, based on characteristics like flexible light-weight technologies, fast and easy assembly operations, temporary occupation of the ground and adaptability to different uses, needs and target groups. Great importance is placed on construction reversibility and environmental sustainability. In the framework of the interdisciplinary research project ‘Urban Pop-Up Housing Environments and Their Potential as Local Innovation Systems’, six scenarios of application for temporary pop-up environments for the city of Vienna have been developed, taking into consideration technical, urban and social aspects, on the basis of local uses and climatic conditions. In order to explore drivers and barriers of the scenarios regarding the transferability of the concepts, online questionnaire sessions were conducted with an international audience. The feedback obtained by the participants allowed an analysis of the applicability of the concepts to other urban environments under comparable conditions at the international level. The paper presents the results obtained from the questionnaire sessions, allowing insight on the international perception of temporary pop-up environments and, specifically, strengths and weaknesses of the scenarios, as well as their possible applicability in the local contexts of the respondents. It was observed that while the perceptions of what requirements temporary housing must fulfil in order to be sustainable are quite uniform among the experts, the identified barriers for implementation within the different international contexts differed greatly. The designs of these temporary housing scenarios, which rely heavily on local resources and systems, are strongly interwoven with the fabric and conditions of the city they were conceptualised for. While this serves to promote the sustainability of these solutions, it poses a particular challenge for the international transferability, requiring heavy adaptation for other contexts.

## Introduction

Cities are characterised by a large built environment that includes various types of buildings and infrastructures, such as streets, bridges and open spaces for pedestrians. The construction, renovation, repair and demolition of these structures require an extensive material input [1] and generate a large waste stream [2] in a one-directional flux of natural resources from the rural to the urban environment, according to the current linear economy paradigm of the ‘take-make-dispose’ plan [3]. Major flows of materials, energy and water are delivered to cities, centres of human and economic activity where more than half of the global population lives, where they are ultimately consumed and/or disposed [4]. As one of the main sectors responsible for global energy consumption and waste production [2, 5, 6], the construction industry plays a critical role in urban development and in rebuilding existing cities in a sustainable way. The sector needs to be aligned to new social, cultural and economic models, keeping incoming products, materials and resources in use, but working out a redesign of their biological and technical cycles, so that their value can be kept at the highest level possible for as long as possible, while preserving natural systems [7].

As part of the transition from a linear to a circular economy, the current construction sector needs to consider new methods and services to create positive environmental impacts and approach circular economy models, which encompass not only production but also processing secondary resources. This requires changing the way products are conceived and allowing the reuse of building components and materials [8], avoiding waste and reducing costs [9, 10]. Nowadays, there are many trends revolving around sustainability in the construction field: waste materials like plastic, wood and rock wool are recycled and used for the realisation of new products [11, 12]; modular manufacturing and prefabricated components allow the construction of modules that are mounted on-site with dry techniques, enabling the possibility of ‘de-constructing’ at the end of their life-cycle for future reuse in new structures [13]; new models of collaboration between the different actors of the different stages of the supply chain (design, procurement, construction) can enable the adoption of circular solutions, favouring a design, oriented towards the disassembly and recovery of materials. But still, new methods and tools are needed [14], as well as new circular approaches that can contribute to the creation of sustainable cities of the future [15], as promoted by the Sustainable Development Goal 11 — Sustainable cities and communities — set in 2015 by the United Nations General Assembly [16]. In this framework, the presented research focuses on temporary pop-up environments (PUEs), designed as innovative models of living systems. Due to characteristics such as light-weight technologies and fast and easy assembly-disassembly operations, PUEs occupy the ground only temporarily, being flexible and adaptable to different uses and target groups [17–21] and, because of their temporary nature, are usually designed for allowing the reuse and after a few cycles recycling of units and components, with advantages in terms of speed and economy, comparing it to a traditional permanent design [20, 22].

The present research paper is part of the 3-year research project ‘Urban Pop-Up Housing Environments and their Potential as Local Innovation Systems’ [23], funded by the Vienna Science and Technology Fund (WWTF). The project aim is to contribute to the transition of the construction sector from linear to circular models, by using temporary PUEs, so as to make urban reuse strategies more sustainable in social, economic and environmental terms, in an attempt to find adaptable and flexible paradigms for different urban contexts. Within the project, innovative models of pop-up living systems are created for the urban context of Vienna, conceptualised as spaces for social learning and experimentation, where new technical and social concepts can be applied and evaluated to explore more sustainable ways of living. Six different scenarios of pop-up housing environments were developed under consideration of different types of urban spaces, which were then further specified into six models.

This paper seeks to explore the international applicability of these scenarios, which were designed for the context of the city of Vienna, using a series of interactive questionnaire sessions which were conducted with international experts. The purpose of the questionnaire sessions was to gain feedback on the pop-up housing scenarios developed within the research project and to gather information about their applicability for the local contexts of the experts, examining the possible drivers and barriers. The first questionnaire session was held during the 2020 Closed Cycles and Circular Society Symposium that aimed to foster the most recent practices, innovations and challenges encountered for closing material cycles in a sustainable way. Following the symposium, further international experts were identified, and the questionnaire session was repeated a total of 4 more times within video conferencing meetings, reaching a total of 18 participants. The expert feedback that was collected allows valuable first insights for the identification of strengths and weaknesses of the scenarios designed for Vienna, as well as insights regarding the international replicability of the scenarios.

## Background

Temporality in construction has remote origins, following a path parallel to humankind’s way of living and interacting with their environment [24]. Temporary structures were one of the first forms of built shelter, with humanity organising as hunter-gather societies for most of human history: over the course of history, they have assumed various forms and characteristics, depending on the specific local context [25]. The concept of ‘temporality’ is in antithesis with that of ‘permanence’, also contrasting with that of ‘architecture’, bound to the classic canons of expression of the durability and permanence of the build, understood both as a material essence and in the historical, cultural and symbolic one. The choice of preferring a ‘temporary’ architecture to a ‘lasting’ architecture may depend on many factors but, in general, will always be based on the assumption that the structure has a limited life, linked to the time in which it will be used, and which, presumably, already includes reasoning about the end of its life cycle [26]. For example, contemporary temporary housing environments have often found their application in emergency contexts as a result of natural disasters, such as earthquakes [27]. During these events, large numbers of people are displaced from their homes which have suffered damage, making them unsafe for living [28]. Moreover, temporary housing is often applied in the case of the emergence of new migratory currents, due to conflicts and climate change [29]. In addition to emergencies, there are also other contexts for which flexible and temporary housing can be appealing, for instance as experimental spaces to explore the future of housing [30]: recently, the global need for temporary housing has increased, and nowadays, the concept is even being implemented in city development projects. It is now common to see temporary pop-up housing projects involving accommodation for students, tourists, workers, migrants and low-income families [22]. During the last few years, public authorities’ attitudes towards temporary uses changed markedly, going from being an obstacle to urban planning to being accepted and even implemented as an urban strategy in certain cases [31]. PUEs have been used as a method of re-appropriation of spaces otherwise cut off from the urban context, with advantages not only from an urbanistic point of view, but also providing social, economic and environmental benefits [21]. There are various examples of pop-up structures built in vacant plots or in empty buildings, realised for a limited period of time until a future permanent destination is identified. Also, PUEs are often used in contexts of densely populated urban areas, where there are various practical and legal limitations due to the rigidity of urban plans and the preservation of historical heritage [30]. The use of PUEs offers the opportunity to study unconventional solutions and experiment with functions, allowing quick and flexible answers and circumventing some bureaucratic planning procedures [21]. However, it must also be said that the ‘construction tradition’ in many countries favours the ‘permanent’ architecture, since the picture of temporary housing as an ‘economic’ construction method slows down its adoption [32]. In addition, while the opportunities which temporary housing provides are becoming more recognised in urban planning, the connotations surrounding temporary housing can still be very mixed, and negative associations are often still prevalent [32]. This is due to examples of temporary dwellings that do not attain adequate standards of sustainability in the social, economic or environmental dimension [33].

It can be noted that, nowadays, the topic of the PUEs embraces a very wide field of applications ranging from emergency situations due to natural disasters or conflicts to the possibility of being used as a temporary urban implementation strategy [21, 22, 27–29, 31]. The range is so wide that it is difficult to define general construction requirements and design criteria, which can be universally valid in the different contexts in which they are created, with the result that, despite in the recent years the debate on temporary PUEs has widened, there is still no universal definition for this term [20]. Taking into consideration the existing sources in the literature, however, it is possible to define some general aspects to achieve sustainable temporary housing, regardless of the context in which the PUEs are implemented and with the aim to achieve social, economic and environmental benefits.

For example, construction speed is a requirement often at the basis of every type of temporary housing, being an important requirement to solve a crisis in a short amount of time and reducing the time related to transportation, construction and deconstruction activities [22, 28, 34, 35]. It is strictly related to the requirement of construction simplicity, such as low structural complexity and easy assembly of the building components, that allows a quick construction and also enables the chance to realise PUEs adaptable to different conditions [35]. The fulfilment of these requirements is strictly connected to the types of materials and components used: the standardisation of components and modules, made by lightweight and prefabricated products, ensures a quick construction of the PUEs [27, 28, 34] and allows the creation of living spaces capable to respond to different functional and spatial needs [27, 32, 34]. Directly linked to the concept of temporariness, it is important to consider the reversibility and sustainability of PUEs, when their temporary use cycle comes to an end. From a technical-constructive point of view, reversibility translates into the possibility of being able to easily and effectively deconstruct the PUE [22, 32], in anticipation of a future reuse or recycling, without leaving indelible marks in the place where the PUEs were constructed, with the aim to reduce the environmental impact [29, 36]. The reuse and recycling of these units also contribute to the reduction of energy consumption and gaseous emissions related to the construction sector [32, 37]. Finally, the people to whom PUEs are addressed are often in a condition of economic hardship, and the economic affordability represents an important requirement for guarantying a housing unit at the minimum costs [22, 32, 35, 38]. Furthermore, it must be said that the current global economic crisis encourages the use of technologies that can be implemented quickly and economically, such as PUEs are [26, 39, 40].

The definition of general requirements for the PUEs, which can be considered regardless of the context of realisation, allows to derive further considerations, like analysing the main factors that lead the planning of temporary strategies. These factors are strongly connected to the type of PUEs considered (e.g. the presence of a crisis situation that requests fast actions), and they can depend highly on geographical and climatic conditions, as well as economic availabilities and political plans. Often, these factors are chosen on the basis of economic and technical considerations (therefore with particular attention to costs related to production, construction, use, deconstruction and storage) [22, 27–29, 36, 38, 41–43], and sustainability (with attention to the environmental impact and the reversibility of the PUEs) [22, 28, 29, 36, 44–47]. But it is also possible to encounter temporary strategies that analyse social aspects, such as the well-being of the users and the aesthetics of the units, with possible implementation of the space according to the personal tastes of its occupants [18, 22, 27–29, 32, 48–50].

Therefore, on the basis of the information available in the literature, it is possible to analyse which are the general requirements and the factors to consider for a sustainable planning of the PUEs. Unfortunately, there is still a lack of information regarding the possible ‘internationalisation’ of the concept of temporary pop-up housing: specifically, this term means the possibility of transferring the PUE to a new urban context, under comparable framework conditions, but taking into account the climatic, environmental, social and cultural and, above all, political and economic differences, which can lead to modifications and adaptations of the original scenario. Although there is an abundance of research regarding the reuse of deconstructed building components of PUEs in new life cycles, or even of PUEs disassembled and reassembled in different geographical contexts, nowadays, the discussion regarding the possible international transferability of the PUEs is still open and needs more insights.

### Scenarios

Six possible temporary housing scenarios were defined within the WWTF-funded research project for the context of Vienna. The scenarios were developed with the aid of stakeholders. Six suitable areas for temporary housing in the city were identified. These are:

1. Vacant lots, understood as urban gaps due to the lack of buildings in densely constructed lots;
2. Empty buildings, like factories and industries that have fallen into disuse and are now abandoned;
3. Large green open spaces (whereby these spaces provide important functions which must be taken into consideration);
4. Rail traffic areas which are no longer in use and the reuse of train wagons for residential purposes;
5. Vacant ground floor retail spaces, before a new destination is found;
6. Bodies of water, like rivers and the reuse of old cargo ships.

Both technical aspects (e.g. architecture, building infrastructure, materials size and use, end-of-life disposal) and social aspects (e.g. possible user groups, duration of stay, adaptability and flexibility for different uses) were defined. The scenarios were developed in a step-by-step process: (1) definition of the scenario (during workshops with interested stakeholders); (2) concept design (in collaboration with the students of the ‘POPUP SHELTER – Design Studio’ course at the Vienna University of Technology); (3) architecture modelling for the definition of the physical 3D models; (4). model assessment for the definition of energy, materials and waste flows. As part of the ongoing project, the six scenarios were at different levels of development, while the questionnaire sessions were being conducted: scenarios #1, #2 and #3 were completely defined regarding concept and architecture and almost completed regarding the model assessment. For scenarios #4, #5 and #6, concepts and architectures were defined, but the model assessment was still ongoing (see Table 1 for scenario summaries). Among the various benefits considered for the selection of scenarios, the reuse of building gaps (scenario #1), empty buildings (scenario #2) and vacant ground floor retail spaces (scenario #5) have the objective to give back to the community spaces which are otherwise cut off from the urban context. There are also positive impacts regarding transport, energy conservation and raw materials, since the structures are already there, and large quantities of new building materials are not necessary. The realisation of PUEs in green and shaded spaces has the objective of finding new and cooler areas of the city to escape heat islands (scenario #3), while the reuse of trains (scenario #4) and ships (scenario #6) allows a second life for the vehicles, the use of otherwise unused spaces and efficient transportation of the housing units to European destinations along the railway or river networks.

**Table 1:** Scenarios summary

**#**	Field of application	Scenario	Implication
1	Vacant lots	GapModule	Temporary units to be realised in urban gaps due to the lack of buildings in densely constructed lots
2	Empty buildings	Life Sharing to Go	Temporary units to be realised in old and abandoned factories and industries that have fallen into disuse
3	Large green open spaces	Beat the Heat	Temporary units to be realised in cooler areas of the city to escape heat islands
4	Rail traffic areas	Life on tracks	Temporary units to be realised in train wagons for residential purposes
5	Vacant ground floor retail spaces	Shop-hopping box	Temporary units to be realised in retail-shops facing the streets that are currently unused
6	Bodies of water	DonAutonom	Temporary units to be realised in old cargo ships moored to riverbanks

#### Scenario #1 — GapModule

**Fig. 1:**
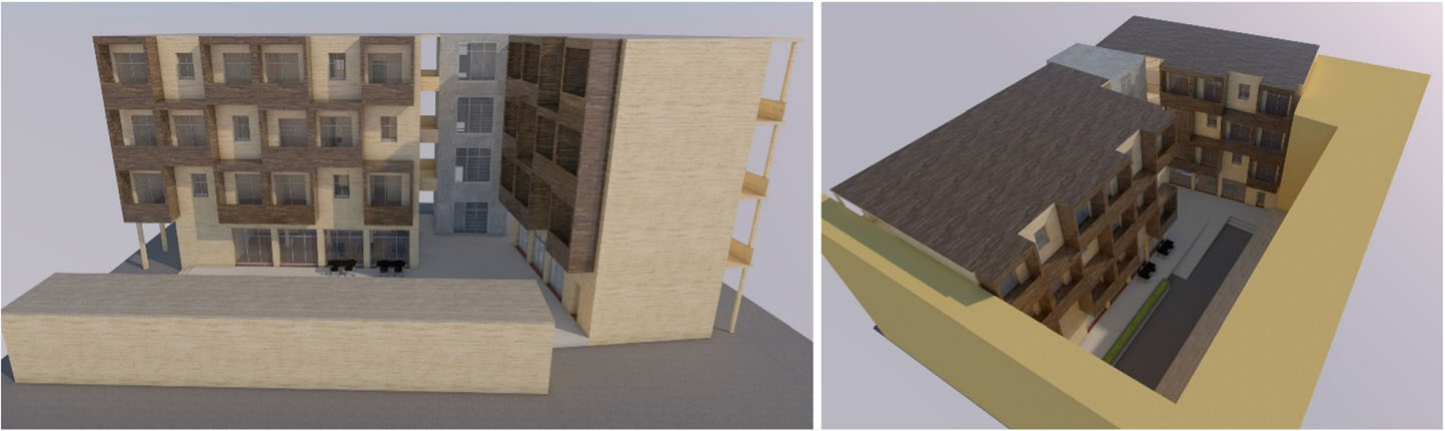
GapModule 3D modelling (design by Friedwagner & Prömpers/Design Studio Pop-Up Shelter/JASEC, TU Vienna/WS 2019, drawings by Gaetano Bertino)

In ‘GapModule’, the pop-up environment is placed in a building gap, a vacant lot between buildings in a residential area, until a new use destination is provided for the area. To adapt easily to the layout conditions of the plot, the temporary structure is made of prefabricated modular components, such as a timber structural frame and timber wall panels, and is designed to endure several mantling and dismantling operations. The foundation structures will also be built with modular prefabricated elements, such as reinforced concrete plinths, made by minimising the use of binders. The modules are transported via trucks on site and are assembled with dry techniques by mobile cranes. Mantling and dismantling operations should take approximately 3 months each, not exceeding a total period of 6 months. The assembled units are used for living for a period not longer than 3 years (maximum period of temporary use before a new permanent destination is found for the building gap) before they are dismounted and placed into storage or are directly reused. In the future, the units can be reused in another building gap, also considering possible maintenance and refurbishment interventions on the unit components that may be necessary over time. When the units are in a condition that does not allow the reuse of components at low refurbishment costs, they would be recycled at the end of their lifecycle. As the plot is part of the urban grid, a connection to the existing water and wastewater, electricity and waste management facilities is assumed. Modular functional green facades and green roofs are applied in order to prevent the increase of urban heat islands, possibly fed by rain- or greywater. This scenario is designed to accommodate a mixed user group of college students and refugees aged between 18 and 30 years, in order to promote social integration. The building provides 35 private rooms, of area between 25 and 50 m^2^, while sanitation and cooking facilities are shared to encourage the collective use among the residents (Fig. 1).

#### Scenario #2 — Life Sharing to Go

**Fig. 2:**
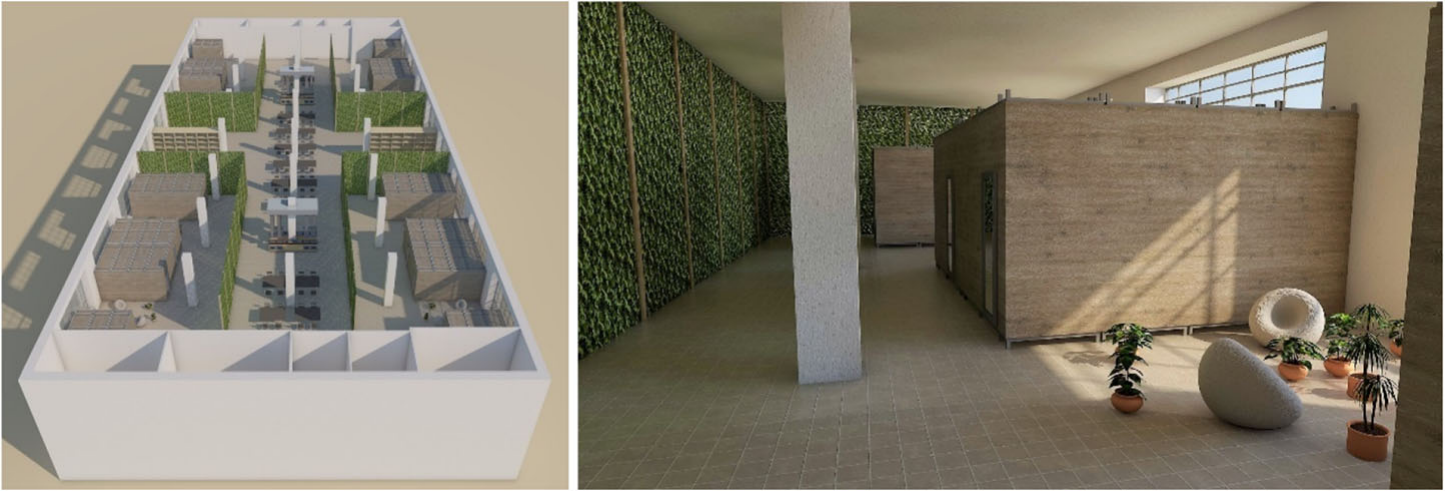
Life Sharing to Go 3D modelling (design by Tasevska & Dimitrov/Design Studio Pop-Up Shelter/JASEC, TU Vienna/WS 2019, drawings by Gaetano Bertino)

The main idea of ‘Life Sharing to Go’ is to implement the concept of communal living within vacant or unused industrial buildings in a residential (not exclusively industrial) area. The indoor spaces of vacant industrial buildings are used for a limited time for the construction of temporary pop-up housing units until the industrial building is given a different permanent purpose. The housing units are realised with the use of prefabricated components and light materials, designed to be transportable as modules by trucks and then assembled with mobile cranes, allowing for fast and easy mantling and dismantling. The housing units are designed to allow further extensions of the modules according to the number and type of users (single, couple, family): starting from a base modular unit, the unit’s dimensions vary between 20 and 40 m^2^. This scenario is meant to accommodate a mixed group of migrants and locals interested in this type of living. The building provides accommodation for 100 people, with private units of area between 15 and 22 m^2^. The bathrooms are private, while cooking facilities and a community garden are shared to promote communication and social interactions. The internal areas of the industrial building which surround the private housing units are also used as common areas for collective use. Depending on the location in the city, it can be necessary to incorporate on-site solutions for water, sanitation services and energy supply. This approach can also possibly use façades and the roof for solar power integration and functional greening purposes, for the case of using wastewater treatment for reuse and/or food provision. A good connection to the public transport system should be provided (Fig. 2).

#### Scenario #3 — Beat the Heat

**Fig. 3:**
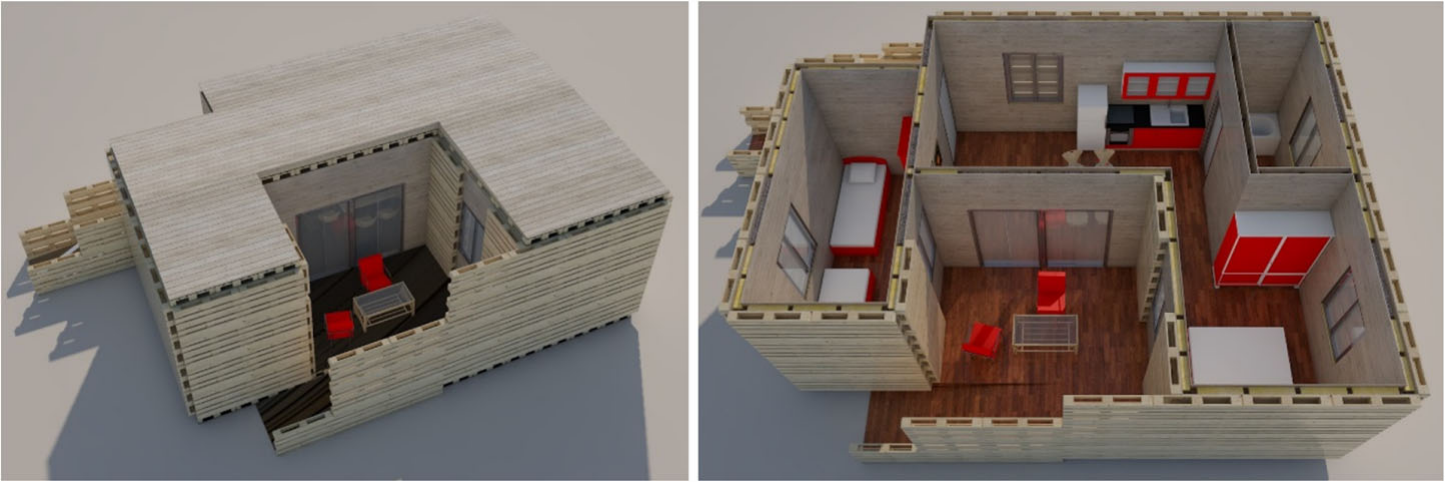
Beat the Heat 3D modelling (design by Barbero & Cuesta/Design Studio Pop-Up Shelter/JASEC, TU Vienna/WS 2019, drawings by Gaetano Bertino)

‘Beat the Heat’ can be described as a pilot study to explore temporary housing systems during heatwaves. It addresses people who are most affected by and vulnerable to heatwaves: senior citizens, families with small children and pregnant women living in accommodations within the city which are particularly affected. This scenario offers temporary accommodation for the duration of the heatwave by temporarily setting up reusable and storable housing in cooler areas of the city, such as open and green spaces at the outskirts. The temporary environment encompasses private housing units for up to 150 people, offering a mix of units for singles, couples and families, with private kitchen and bathroom facilities and accessibility for wheelchair users, with private units with an area between 30 and 50 m^2^. The temporary housing structures are assembled at the beginning of each heatwave and disassembled once the temperatures decrease, with the substantial features of quick and easy mantling and dismantling, transportation and storage for future use. The units will be realised with reusable materials, such as timber for structural frame, wood for non-structural elements and wooden shallow foundations. The residents use the housing units for only a few weeks at a time, but the housing units themselves are expected to endure several years in the context of assembly-use-dismantling-storage every season. The housing units have a cool and comfortable indoor climate through the use of passive cooling systems, such as ventilated walls that allow the least possible use of energy and evaporative cooling. The area above the buildings can also be shaded through the use of sunsails (Fig. 3).

#### Scenario #4 — Life on Track(s)

**Fig. 4:**
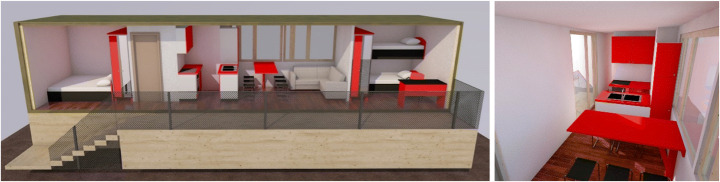
Life on Track(s) 3D modelling (design by Neudeck & Werni/Design Studio Pop-Up Shelter/JASEC, TU Vienna/WS 2019, drawings by Gaetano Bertino)

‘Life on Track(s)’ is a scenario in which living space is provided in wagons on tracks that can be moved rapidly from one site to another without organisation of additional carriers. In this way, in a short period of time, a large amount of temporary housing units can be transported and placed, simply by taking advantage of the large number of tracks in and between cities. In order to be suitable for housing, the final location of the wagon units must be in an area that can meet the needs of the residents concerning connectivity in the city and infrastructure. The starting point for the living spaces are gutted passenger carriages, couchette carriages and flat wagons on which a living environment is created, for a total liveable area of 25 m^2^ per private unit. In the latter case, containers can be used on the flat wagon, allowing the opportunity to use the housing units even off the tracks and using folding and expandable platforms that create additional space. Regarding the water supply and the sanitation system, on-site solutions have been considered. Solar thermal collectors integrated in the wagons or additional locomotives attached to the wagon units can be equipped to the unit to provide energy supply. On-site solutions, like photovoltaic panels on the wagon roofs, can be utilised. The planned period of residence of the users spans from a few nights up to several weeks, and the potential user groups range from people affected by homelessness who can be homed during cold seasons to festival respondents and tourists (Fig. 4).

#### Scenario #5 — Shop-Hopping Box

**Fig. 5:**
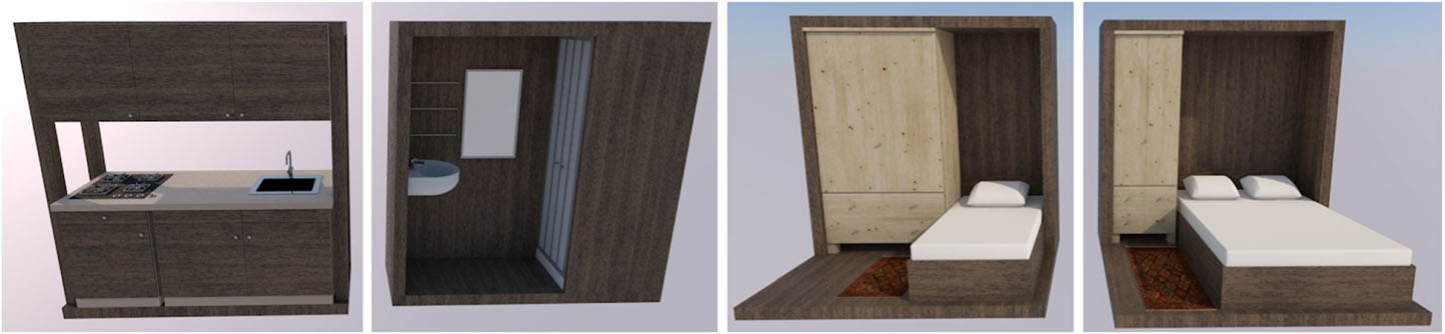
Shop-Hopping Box modelling (design by Pelaez & Rodriguez/Design Studio Pop-Up Shelter/JASEC, TU Vienna/WS 2019, drawings by Gaetano Bertino)

‘Shop-Hopping Box’ addresses the problem of high vacancy rates for ground floor retail spaces, especially when they are not situated in prime shopping streets. To make better use of this available built environment, these vacancies are temporarily appropriated as living spaces until another retailer moves in, with the use of pop-up living units realised off-site and mounted on site with dry techniques that allow easy and fast mantling and dismantling operations. The units are made of flexible and modular prefabricated components for the application in different architectural frameworks or floor plans. They are designed complete with modular home furnishing, including mobile kitchens and wet cells that are easily assembled and disassembled and fit through available doors and windows of the retail spaces, and allow space-saving storage, when not in use. This scenario aims to house individual people, and small families who voluntarily choose to participate in this rather unconventional form of housing and are in need of living space for a limited time period not exceeding 24 months (Fig. 5).

#### Scenario #6 — DonAutonom

**Fig. 6:**
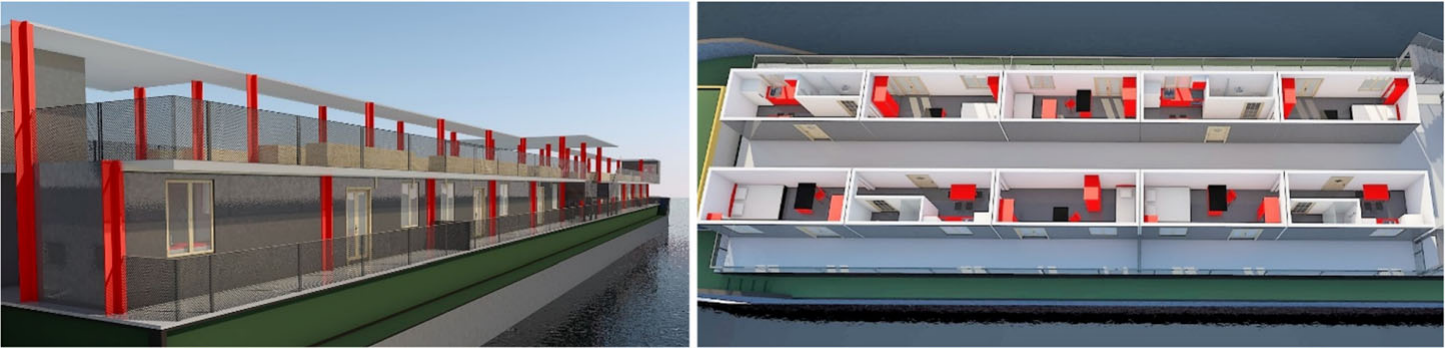
DonAutonom 3D modelling (design by Dembski & Wössner/Design Studio Pop-Up Shelter/JASEC, TU Vienna/WS 2019, drawings by Gaetano Bertino)

‘DonAutonom’ involves the use and redesign of old cargo ships that can be purchased, with the idea of being anchored on the coast to offer a short-term home for different types of user groups. ISO containers are used as temporary units, providing a liveable area between 25 and 40 m^2^. Also, the interior of the ship is converted into attractive common spaces. Corrective measures, which also concern the modification of parts and components of the ship's bodywork, are realised to remedy the limits of lighting and ventilation. The strong point of this concept is the high degree of autonomy or self-sufficiency in the use of resources: rain- and river-water can be reused for irrigation in the common green areas and flushing toilets, and biogenic waste and faeces can be converted into biogas, to be then used as an alternative mode of propulsion for short distances by ship. Since ships offer a lot of conversion and remodelling potential, the possibility of a year-round supply of seasonal food is considered and can be addressed by gardening systems on board, possibly with the use of greenhouse systems. The ships are planned to be anchored in place for about 3 years before changing their position to another location. Anchor points are chosen to be in close proximity to the underground trains, in order to allow good connections with the city. This concept of housing can accommodate people who live in the city for a short term, such as seasonal workers, students and tourists (Fig. 6).

## Methodology

### Questionnaire Sessions

As stated in the introduction, the present paper aims to explore the international applicability of these scenarios, which were designed for the Viennese context, and to gain insight on possible drivers and barriers, so as to understand their strengths and weaknesses. For this purpose, the authors took part in the 2020 Closed Cycles and Circular Society Symposium, hosted by the Zurich University of Applied Science ZHAW and the International Ecological Engineering Society IEES on 2–4 September 2020. The symposium was planned to be held in Zurich (Switzerland) but due to COVID-19, it was held online. Subject matter were the most recent practices, innovations and challenges encountered in this field, with the aim of developing new approaches. The target groups were experts and practitioners, such as scientists, planners, architects and engineers, who have an interest in the transition towards a circular society and international discussion. The authors presented the case study session ‘T/CS3.4 - Buildings as ecosystem services providers - Drivers and barriers for implementation and transferability of sustainable temporary pop-up living systems’, open to all of the symposium’s participants with an interest in the topic. The workshop had to be carried out online, resulting in the design of an experimental interactive questionnaire session. The questionnaire session, lasting an hour and a half, was structured in a way which allowed a continuous exchange with the participants, following an introductory input from the presenter. The polling app ‘Slido’ was utilised, an easy-to-use Q&A and polling platform for live or remote meetings, events, classes and webinars. Within the questionnaire session, participants could answer the questions displayed on the screen with a simple Slido event code and/or link. The respondents answered in a synchronous manner during the session, with the results being transferred instantly. With the Slido tool, event organisers can moderate incoming questions, so they have full control over what is being displayed on the presentation screen, as well as activate polls [51]. This format allowed respondents to ask questions of understanding before entering their answers and engage in brief exchanges surrounding the questionnaire topics. The questionnaire consisted of 7 parts, with an alternance between the presentation (e.g. including the description of the 6 temporary housing scenarios) and the questionnaire, with space for discussion between the parts.

After the positive experience with the interactive online questionnaires at the 2020 Closed Cycles and Circular Society Symposium, the session was repeated with interested experts who were selected and invited by the authors, chosen specifically to represent a broader range of countries (therefore also different geographical and climatic characteristics) and to represent varying fields of knowledge and expertise according to their occupations. In a total of 5 sessions, 18 experts participated, representing 11 different countries from 4 continents.

### Questionnaire Content

The session began with a presentation of temporary pop-up environments as innovative models of living systems, approaching circular economy models from production to processing waste. The ongoing research project and the six scenarios developed for the city of Vienna were then presented, including considerations on the technical, urban and social aspects, as well as local uses and climatic conditions, that characterise them.

The ‘questionnaire blocks’ were presented in stages, as the various topics emerged: block #1 concerned general information on the respondents, including occupation, country of residence and experience with temporary housing; block #2 was about the respondents’ positions regarding the requirements that temporary pop-up environments should have; block #3 concerned the respondents’ opinions on which factors were most important when planning a temporary housing strategy; blocks #4, #5 and #6 concerned, respectively and in detail, assessments of the scenarios ‘GapModule’, ‘Life Sharing to Go’ and ‘Beat the Heat’; block #7 concerned the scenarios ‘Life on Track(s)’, ‘Shop-Hopping Box’ and ‘DonAutonom’ and final considerations.

The questions for the sessions were selected and developed in the months prior to the symposium, based on interdisciplinary discussions between the project members. The questions are made up of multiple-choice and open-ended questions. In this way, it was also possible to obtain clarifications and insights on the responses of the participants. The first questions aimed to obtain information regarding both the expertise of the participants (so as to have information on their experience on the subject and the expectations they place on the subject based on their professional role) and the topic of temporary PUEs (in order to obtain an overview of which criteria and requirements are considered most important to achieve successful temporary strategies). The questions then went into the specifics of the Viennese scenarios, with the aim of obtaining feedback regarding their strengths and weaknesses in order to better analyse how to implement these solutions in international contexts. Since the questionnaires were interactive, it was possible to resolve questions of understanding or comments immediately, to obtain more detailed remarks regarding the answers and to bring the audience closer to the topic and the project.

### Questionnaire Analysis and Limitations

The questionnaire provides both quantitative data and elaborations gathered through open questions. The quantitative data primarily serves to complement the detailed considerations with a broad overview of tendencies. Where applicable, the ‘mean’, ‘median’, ‘maximum’ and ‘minimum’ values of the ratings are presented for this purpose. While the mean describes the average value (total of numbers divided by how many numbers there are), the median describes the middle value (the number which is in the middle) [52]. Correlations were investigated for question blocks #2 and #3 regarding the positions on requirements of temporary pop-up environments and the most important factors when planning temporary housing, to examine whether any relationships could be identified.

It must be stressed that when regarding the quantitative data, the number of respondents is very low and therefore cannot be regarded as representative. They only serve to provide a first explorative overview and complement the qualitative data. The correlations in particular must be regarded critically, serving only to scan for potential areas which could be of interest for future investigation. The low number of respondents stems from the format that was chosen, with greater importance having been placed on the interactivity of the questionnaire sessions and the qualitative data, which was deemed to be a more effective way of understanding the contexts and reasoning behind the assessment of the applicability of the models from the international respondents.

Due in part to the fact that the 2020 Closed Cycles and Circular Society Symposium was meant to take place in Switzerland and mainly addresses a European audience, European and especially Swiss experts and practitioners were overrepresented. In addition, the number of respondents with direct experience with temporary housing is relatively low, though it is surprisingly high when considering the fact that the participating experts were primarily recruited from a conference and network surrounding circular economy. Practitioners with experience with temporary housing had already been involved in the scenario development phase at an earlier stage of the project.

## Results

### Background Information on Respondents

**Fig. 7:**
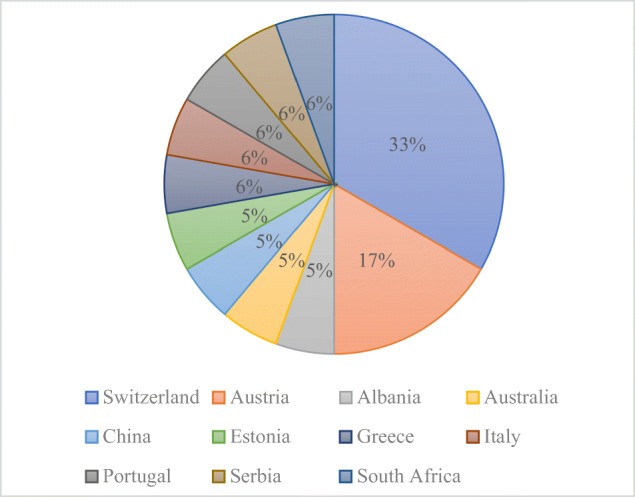
Respondents’ country of residence chart

The respondents came from 11 different countries from 4 continents (Albania, Australia, Austria, China, Estonia, Greece, Italy, Portugal, Serbia, South Africa, Switzerland), with 15 respondents being European (Fig. 7).

**Fig. 8:**
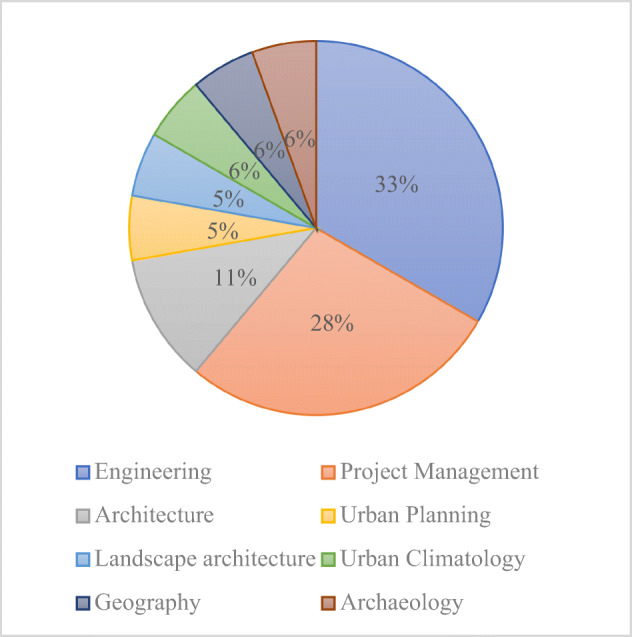
Respondents’ role in their company chart

The following roles were represented: civil and environmental engineers (6 respondents), general managers (5 respondents), architects (2 respondents), an urban planner, a landscape architect, a geographer, an urban climatologist and an archaeologist. The topic of ‘temporary housing’ was of great interest for participants from different professions who regarded the subject from different perspectives. The interest of the urban climatologists, for instance, primarily stemmed from the positive impact of temporary homes on the environment, thanks to the recovery and reuse of units and components, directly and indirectly affecting the urban climate. In the case of the archaeologist, the interest in PUEs was explained by the need to use lightweight structures on archaeological sites, with the aim of preserving the soil on which they are built as much as possible and allowing quick assembly and disassembly activities. Thirteen of the respondents have never been directly involved in temporary housing projects, three participants had up to 2 years, one participant between 2 and 5 years and one participant over 10 years of direct experience with temporary housing projects (Fig. 8).

### Requirements for Temporary Housing

The second questionnaire block concerned the respondents’ positions regarding the requirements that temporary environments should fulfil. Respondents rated pre-defined requirements to evaluate their importance on an increasing scale from 1 to 5 (least importance to most importance) (Table 2).

**Table 2:** Comparison table for question block #2 (n = 18)

Variable Nr.	Requirement	Mean	Median	Min	Max
V1	Modular components	4,3	5	2	5
V2	Demountable, storable and reusable	4,6	5	4	5
V3	Lightweight and easy to transport	3,9	4	1	5
V4	Fast and easy in assembly and disassembly	4,1	4,5	1	5
V5	Adaptable to different uses and target groups	4,2	4,5	1	5
V6	Leave no marks and preserve the quality of the ground	4,1	5	1	5
V7	Recyclability of components	4,7	5	3	5
V8	Affordability	4,3	5	1	5

In general, the scores for each requirement are quite high and they were globally considered important aspects for the realisation of PUEs and temporary housing strategies. The highest ratings were achieved by V7 ‘recyclability of components’ with an average score of 4,7 — with every respondent giving it a rating of 5, with the exception of two individuals who rated it as 4 and one who gave it a rating of 3 — and the aspect V2 ‘demountable, storable and reusable’ with an average rating of 4,6, having been exclusively rated as 4 or 5 by all respondents. Waste is a big problem in the construction sector, and many respondents addressed this during the session. The chance to recycle building components and products seems to be the most important requirement for the participants, which must be taken into consideration when planning temporary housing projects. This could be an area where temporary housing can make a great contribution to the building sector as a whole, experimenting with reusability and recyclability of components.

These aspects are followed closely by V1 ‘modular components’ and V8 ‘affordability’ (whereby affordability refers to the costs for the construction, transportation, running and dismantling/recycling processes), both having an average score of 4,3 and a median of 5. Engineers and architects tended to rate V1 positively, underlining the advantages of building with modular components.

The aspect V5 ‘adaptable to different uses and target groups’ received an average score of 4,2, with the ratings ranging from 1 to 5. The rating of this aspect could be particularly dependent on the main objectives of the temporary housing projects. The reasoning behind one of the low ratings is related to the possible obstacle for the future reuse in new contexts. The concern is that the difference in culture and religion would require too many adaptations. The lower scores can also simply reflect that temporary housing can have many different aims, including experimenting with new materials or aesthetics, which would not necessarily be related to an adaptability to different uses and target groups.

The aspects V4 ‘fast and easy in assembly and disassembly’ and V6 ‘leave no marks and preserve the quality of the ground’ are both tied with an average score of 4,1, whereby the latter has a median of 5, as opposed to 4,5. Assessments regarding the importance of these aspects appear to show a varied picture, spanning the entire spectrum. Regarding V4, this aspect may increase costs if it is not given (by requiring expert assembly and disassembly). The rating of V6 likely varies according to contextual factors, perhaps being of importance when on a green field, but less relevant when in a building gap or within a building.

V3 ‘Lightweight and easy to transport’ received the lowest average score of 3,9, despite almost half of the respondents having given it a rating of 5. This aspect also has the lowest median value with 4. It is possible that the ratings may vary according to contextual factors. V3 is related to V2, which got high ratings across the board. For temporary housing to be demounted, stored and reused, transportation will usually be part of this process. If this factor V3 is considered not to decrease the factor of V2, then the lower rating could imply that the costs for transportation (in terms of money and time) should not be a prioritised element in the conceptualisation. This may be dependent on the duration of stay: for example, if the transport is only necessary once every 3 or 5 years, it is much easier to cover the costs than if the transport has to be organised every few months. There would likely be a noticeable impact on reusability if transport is very difficult and expensive. Concerning the aspect of being ‘lightweight’, respondents contested that a building made out of light materials cannot give the same feeling of safety that traditional buildings (made out of heavy materials, such as concrete) usually provide, especially after natural disasters (such as earthquakes); another participant pointed out that it is possible to have easy dismounting processes with heavy materials like prestressed concrete panels, produced in a factory and ready to be mounted.

### Relevance of Factors for Temporary Housing

The third questionnaire block concerned the respondents’ opinions on which factors were most important when planning temporary housing. Respondents were asked to rate pre-defined requirements, in order to evaluate their importance in an increasing scale from 1 to 5 (least importance to most importance). The respondents were also allowed to specify their answers in the form of open questions (Table 3).

**Table 3:** Comparison table for question block #3 (n = 18)

Variable Nr.	Factor	Mean	Median	Min	Max
V1	Total expenses	3,8	4	2	5
V2	Space required for storage	3,4	3	2	5
V3	Space required for implementation	3,8	4	2	5
V4	Aesthetics of the solution	4,0	4	3	5
V5	Lead time for temporary housing	3,8	4	2	5
V6	Environmental impact	4,6	5	2	5
V7	Well-being of users	4,3	5	1	5
V8	Reversibility	4,2	4	1	5

In general, all of the factors were considered as being important by the respondents. The factor V6 ‘environmental impact’ achieved the highest average score with 4,6, with a clear distance to the second-highest average score of 4,3 for V7 ‘well-being of users’. Both V6 and V7 are the only aspects with a median of 5 and share a similar distribution of ratings. The high rating of V6 emphasises the potential of temporary housing for sustainable building. However, this score also reflects the concern that through their temporary nature, temporary housing could be the source of much waste, so the environmental impact must be given particular consideration during planning. The fact that V7 garnered the second-highest average score shows that temporary housing is not just regarded as providing temporary shelter from the elements with the purpose of covering the most essential needs, but that it is regarded as ‘housing’ in a sense of homing people and providing comforts able to cover human needs beyond the basic needs ensuring survival.

V8 ‘reversibility’ received the third-highest score with an average of 4,2 and a median of 4. The overwhelming amount of high ratings by most respondents indicate that V8 can be considered a core characteristic of temporary housing. The rather uniform rating among the participants is quite interesting when regarding it compared to the very varied ratings the related aspect ‘leaves no marks and preserves the quality of the ground’ received as a requirement for PUEs in the earlier question block #2. This may be due to ‘reversibility’ being broader in scope.

V4 ‘aesthetics of the solution’ received an average score of 4,0, with the ratings 5, 4 and 3 having been chosen equally often. Not a single participant answered with a score of 2 or 1, displaying a uniform agreement that aesthetics have a place in planning temporary housing, which is even prioritised (on average) over V1 ‘total expense of production (de-)construction, running costs and storage’, V2 ‘space required for storage’, V3 ‘space required for implementation’ and V5 ‘lead time for temporary housing’. Three aspects are tied with an average score of 3,8, namely V1, V3 and V5. There is a strong agreement that total expense is an important factor to consider, with only one person rating it below 3. The aspect V3 sees a distribution of answers almost equally spread over ratings of 5, 4 and 3, with only one individual rating it as 2. Urban planners in particular appear to have rated this aspect highly. The even distribution between the scores 3 and 5 indicates that space is an important factor, but it is perhaps not so scarce as to be considered a higher priority. The third tied aspect V5 ‘lead time for temporary housing’ appears to be an important factor for the planning phase, but on average scores below factors such as V6, V7 and V8. The lowest rating was received by V2 ‘space required for storage’ with an average rating of 3,4 and a median of 3. This is the only aspect which received on average more neutral or negative ratings than positive ones. The importance of V2 is highly contextual (frequency and duration of storage need), and different assumptions can be made regarding the availability or costs of storage

In the open comments, the respondents introduced various additional aspects, such as location, access to electricity and water/sanitation, affordability for the end-users, integration into the local, cultural landscape and waste production. These are all elements which are addressed within the WWTF-project, confirming their relevance (also at the international scale).

### Strengths and Weaknesses of Pop-up Scenarios

The fourth, fifth and sixth parts of the questionnaire concerned the three scenarios ‘GapModule’, ‘Life Sharing to Go’ and ‘Beat the Heat’ and specific questions regarding their respective strengths and weaknesses and applicability for international implementation. The participants rated each scenario on a scale from 1 to 5 (from least to most positive) and provided feedback about which aspects they considered especially positive in the scenario (‘adaptability’, ‘modularity’, ‘reusability’, ‘easy mounting and dismounting’, ‘lightweight’, ‘shared spaces’ and ‘other’), with the option of formulating open answers to expound their choices. Feedback was also gathered for each scenario regarding possible drivers for implementation into the local frameworks of the respondents, as well as barriers and challenges (‘legal’, ‘social’, ‘political’, ‘space constraints’, ‘economical’, ‘environmental’, ‘other’), again with the option of complementary open answers. Lastly, the respondents were asked if they could envision the respective scenario in another city aside from Vienna, with the option of elaborating in an open question format. All questions were answered by all 18 respondents with exception of rating the three scenarios (n = 15) and naming a particularly positive aspect for the scenario ‘Beat the Heat’ (n = 16) (Table 4).

**Table 4:** Rating comparison table (n = 15)

Scenario	Mean	Median	Min	Max
Scenario 1# — GapModule	3,9	4	3	5
Scenario 2# — Life Sharing to Go	3,9	4	2	5
Scenario 3# — Beat the Heat	3,7	4	1	5

#### GapModule

‘GapModule**’** was given an average rating of 3,9. Four of the respondents (27%) gave it a rating of 5, six rated it as 4 (40%) and five as 3 (33%), making the distribution relatively even across these three ratings. Nobody rated this scenario as 2 or 1. GapModule was well-received by the respondents (Table 5).

**Table 5:** Which aspects do you consider specifically positive in this scenario? (n = 18)

Driver	Nr. of times chosen	Percentage
Adaptability	12	67%
Modularity	10	56%
Reusability	12	67%
Easy mounting and dismounting	10	56%
Lightweight	5	28%
Shared spaces	9	50%

Regarding the positive aspects in this scenario, ‘adaptability’ and ‘reusability’ were considered especially positive by most respondents, followed closely by ‘modularity’ and ‘easy mounting and dismounting’. Half of the respondents chose ‘shared spaces’. Only four respondents considered ‘lightweight’ as an especially positive aspect of this model. Regarding this point, it was argued that this scenario does not look temporary, and it involves the use of heavy materials for the structural parts. The ‘tactical use of the vacant spaces’ was emphasised as particularly positive in the open comments, while possible problems of integration between different user groups due to differing needs and schedules were also noted as a potential problem (see Table 6).

**Table 6:** What do you think are barriers and challenges for the implementation in your local context? (n = 18)

Barrier	Nr. of times chosen	Percentage
Legal	7	39%
Social	8	44%
Political	2	11%
Space constraints	2	11%
Economical	2	11%
Environmental	2	11%
Other	0	0%

Regarding the possible drivers for implementation, a recurring theme was the driver of very limited affordable living space in dense cities. These answers stem primarily from Austria and Switzerland but were also brought up by respondents from Greece and Kosovo. In total, seven answers related to the issues of limited vacant spaces or affordability of housing. Three responses from Serbia, Italy and Portugal stated that there are available unused building gaps which could be utilised in this way. A respondent from South Africa noted the prerequisite of ‘matching national building regulations and municipal by-laws’. Economic drivers are named in three answers by respondents from Switzerland, China and Albania. Some responses named specific user groups as drivers, with refugees being explicitly named in three answers from Australia, Austria and Greece; students being named in three responses from Serbia, Austria and Greece. Immigrants were also named in a response from Austria, and seasonal workers and those affected by natural disasters were named in a response from Australia. An answer from Switzerland mentioned the (cheap) implementation of welfare support structures as a driver for public authorities. One respondent from China spoke of ‘experimental urban projects’, recognising experimentation as a possible driver for temporary housing. One response from Estonia stated that the scenario does not fit into the Estonian context, elaborating this further under the segment on barriers and challenges.

Regarding barriers and challenges for implementation, those most often identified by the respondents were ‘social’ and ‘legal’, with them being chosen seven times each.

The elaborations given for the ‘social’ aspect were very diverse. Three responses voiced concerns over acceptability and the response from neighbours, with one of these focusing on aesthetic or safety aspects (Australia), one referring to possible noise pollution (Austria) and one mentioning concerns about the acceptance by neighbours (Switzerland). A respondent from Portugal voiced the concern that the conceived user mix might have low acceptance. These responses are unsurprising, seeing as this mode of living is directed at a very specific group of individuals who are open to this integrated kind of community living, and is not aimed towards the average citizen. The Estonian respondent who had earlier stated this scenario does not suit the Estonian context elaborated that community living is not culturally present in Estonia, and that refugees are also not a large user group, due to political reasons. This respondent also pointed out that affordability of housing is not really an issue in Estonia, so there is no high demand for temporary solutions. The timespan of the model was mentioned in two responses, with a respondent from Kosovo finding it too short for social integration, considering that community projects may be more appropriate for the limited available space. The respondent from Australia stated that ‘if this space is meant to be used for more than a few months, fewer people are likely to perceive it as desirable due to it’s a temporary notion’, which appears to imply the planned duration being too long. This respondent was also the only one to mention the environmental aspect, calling into question whether temporary solutions can be more environmentally friendly than permanent residences and if this can be communicated effectively.

The elaborations for the ‘legal’ aspect address that legislation can be strict (Switzerland) and that these processes function on a different time-scale than would be required for temporary housing, with the processes being lengthy and not being suited for short-term licenses (Greece and China), legislation being complicated (China), the legal aspects being interwoven with political aspects, such as political will (China), and property relations are mentioned in terms of rights of private land owners (Switzerland). The fact that anything ‘unconventional’ is difficult for legal aspects is also mentioned (Austria).

The elaboration of the ‘space’ aspect includes a response from Switzerland stating that space is limited and very expensive. As already mentioned above, a response from Estonia noted the opposite for their country of residence that enough affordable living space is available. Interestingly, both the lack of space and the lack of need for space are considered barriers. A respondent from Switzerland also mentioned an environmental aspect, noting that unused space gives room for many other species (flora and fauna), which would be an argument against a re-use of these spaces.

The elaboration of ‘environmental’ includes a comment that sewage and water are not universally available in developing countries (South Africa). This means that the model requires off-grid solutions in these cases. A respondent from Australia questioned if this model can be more sustainable than permanent housing. The argument of open spaces catering to flora and fauna was brought up under the aspect ‘space’ but is of course also an environmental issue.

The elaboration of ‘economical’ mentions that building gaps can be very large, which goes hand-in-hand with expenses (Italy). The need for political goodwill by the local government is also brought up (Serbia). The ‘political’ aspect includes the fact that certain user groups, such as refugees, are not always present (see comment from Estonia), that anything ‘unconventional’ can be a great barrier (Austria), and that a political will is required (Serbia and China) (Table 7).

**Table 7:** The present scenario was developed for the context of the city of Vienna. Can you imagine this scenario in another city? (n = 18)

Answer	Nr. of times chosen	Percentage
Yes	15	83%
No	0	0%
Not sure	3	17%

Regarding the possibility to develop this scenario in a context other than the city of Vienna, 15 of the respondents answered with ‘yes’, nobody answered with ‘no’ and three answered with ‘not sure’. In general, many respondents appear to be able to envision this model for bigger cities in central Europe. Cities and areas mentioned by name are Berlin, Rome, Madrid, Zurich (named two times), Milan, Copenhagen, Lille, Brussels, Wädenswil, Munich and Warsaw. Answers also included ‘any large city in Central Europe with a tight market for affordable apartments’, ‘many cities in Portugal’, ‘many, as long as the appropriate local approvals are obtained’, ‘any other city with similar gap spaces’, ‘any growing city’, ‘many regional cities in Australia that have a seasonal population and regions that are prone to natural disasters’. The suitability for students is named twice by respondents from Switzerland and Austria.

#### Life Sharing to Go

‘Life Sharing to Go’ was given a rating which averaged 3,9. Five respondents gave it a rating of 5, six respondents rated it as 4, two as 3 and two as 2 (Albania and Kosovo). Life Sharing to Go was generally received well by the respondents. While it scored the same as GapModule, the ratings differ in their distribution. More respondents deemed Life Sharing to Go to be ‘very good’, but at the same time, more respondents also had a more critical view.

Regarding the positive aspects in this scenario, ‘adaptability’ was considered especially positive, followed by ‘easy mounting and dismounting’ and ‘shared spaces’. ‘Reusability’ was considered a positive aspect by more than half of the respondents, while ‘modularity’ and ‘lightweight’ were chosen least often (see Table 8).

**Table 8:** Which aspects do you consider specifically positive in this scenario? (n=18)

Driver	Nr. of times chosen	Percentage
Adaptability	13	72%
Modularity	7	39%
Reusability	10	56%
Easy mounting and dismounting	11	61%
Lightweight	4	22%
Shared spaces	11	61%

Regarding possible drivers for implementation, the most mentioned driver was the presence of unused industrial buildings and the idea of reusing or revitalising these spaces (Kosovo, Serbia, Australia, Switzerland, Greece, Portugal). This was followed by the need for affordable housing, named by four respondents (Switzerland, Albania, Estonia). Related to this, one respondent named the scarcity of available housing (Switzerland). Some respondents mentioned specific user groups who could need this type of housing, such as ‘people in need’ (Serbia), ‘the young, not so wealthy people, such as students’ (Estonia), ‘seasonal workers’ and ‘employees of large companies’ (China). Factors relating to changes in urban population, migration and integration were mentioned by respondents from Kosovo and Austria. Drivers which were only mentioned once were ‘economic benefits’ (Albania) and the contribution of ‘creativity and inspiration for occupants’ (Australia). One respondent pointed out that there are municipal by-laws and limits per building according to the National Building Regulations (South Africa), which relates to barriers and challenges. One respondent used the option to specify other aspects, noting ‘big spaces for larger number of people’ (Table 9).

**Table 9:** What do you think are barriers and challenges for the implementation in your local framework? (n = 18)

Barrier	Nr. of times chosen	Percentage
Legal	9	50%
Social	5	28%
Political	4	22%
Space constraints	0	0%
Economical	3	17%
Environmental	0	0%
Other	2	11%

Regarding barriers and challenges for the implementation, the ‘legal’ aspects were identified by the most respondents (a total of nine) as being a barrier or challenge for implementation. Three of the responses pointed to the issue of needing the building owner or site owner to be on board with the realisation of such a project (Portugal, Austria, Italy), noting that incentives would be required. A respondent from Estonia stated that the housing must be affordable in order to be an option in Estonia, and that already existing artistic ‘cultural centre’ projects in privatised post-industrial areas are perceived as being exclusionary. The fact that the industrial sites in this model are also privately owned may be a concern for this reason. As with the last model, the need for government support is named (Serbia). One of the respondents also pointed to COVID-19 making the implementation of shared social spaces difficult (South Africa).

Regarding ‘social’ aspects, five respondents deemed this to be a barrier or challenge for implementation. Acceptance by locals for these kinds of social spaces is brought up by three respondents (Serbia, Switzerland), with a respondent from Switzerland explicitly pointing to the rather reserved nature of the Swiss. Privacy and safety are also named by a participant from Australia as being key challenges. A respondent from Kosovo questions, whether this short time span can even achieve the goal of social integration.

Regarding ‘political’ aspects, four respondents chose this as an important barrier. The comment of the respondent from Estonia regarding the ‘cultural centres’ in privatised post-industrial areas also applies to this dimension. The comment, that politics of affordable housing must be used in this scenario, also applies here. The fact that site owners or building owners need incentives to grant access and use of their properties is also a political question which can be discussed in this context, as is the need for government support. A Swiss respondent states they can imagine that the use of these buildings for non-industrial or non-business purposes could trigger a political discussion.

Three participants chose the ‘economical’ aspect, with a participant from Switzerland pointing out that the maintenance of the abandoned building must be maintained, and investments must be made in this regard. A participant from Greece argued that noted that this model could be suitable for art residents or students. They also point out that this model could be suitable for housing refugees. The third participant who chose this aspect is from Estonia, noting that this type of housing needs to be affordable to be applicable for their country. Nobody chose ‘space constraints’ or ‘environmental’ as important barriers or challenges for the implementation of this project in their local framework. A participant from China mentioned that in their country industrial establishments are usually far from the city, creating a problem of accessibility (Table 10).

**Table 10:** The present scenario was developed for the context of the city of Vienna. Can you imagine this scenario in another city? (n = 18)

Answer	Nr. of times chosen	Percentage
Yes	15	83%
No	1	6%
Not sure	2	11%

Regarding the possibility to develop this scenario in a context other than the city of Vienna, 15 of the respondents answered with ‘yes’, one respondent answered with ‘no’ and two answered with ‘not sure’. Cities and areas mentioned by name are South Italy, London, Novi Sad, Podgorica, Szeged, Berlin, Warsaw. The responses were more general for this example, with most respondents envisioning it for any large cities in Central Europe, with several also stating that they can imagine this being applied anywhere in the world, in post-industrial areas which are close to the city, or gentrified neighbourhoods with old warehouses. One respondent from Austria can even imagine this solution for smaller towns. A respondent from Greece mentions that Greece has large islands where industries used to be operated and are now abandoned.

#### Beat the Heat

‘Beat the Heat’ was given an average rating of 3,7, scoring 0,2 points lower than the other two scenarios. Four of the respondents gave it a rating of 5, five rated it as 4 and four as 3. One participant rated it as 2 and one person as 1. While Beat the Heat was generally received well by the respondents, two rated it poorly (Switzerland, Austria), and two respondents also abstained from choosing an aspect they find particularly positive about the scenario. This reflects that this scenario did not resonate with all respondents, with some viewing it quite critically (Table 11).

**Table 11:** Which aspects do you consider specifically positive in this scenario? (n = 16)

Driver	Nr. of times chosen	Percentage
Adaptability	8	50%
Modularity	7	44%
Reusability	12	75%
Easy mounting and dismounting	11	69%
Lightweight	8	50%
Shared spaces	4	25%

Regarding the positive aspects in this scenario, ‘reusability’ and ‘easy mounting and dismounting’ were considered especially positive, followed by ‘lightweight’, ‘adaptability’ and ‘modularity’. ‘Shared spaces’ was chosen least often, reflecting the fact that this was not a strong focus of the scenario. The elaborations in the comments praise the fact that climate vulnerability is addressed (Australia), the inclusion of especially vulnerable age groups (Estonia), and the fact that the scenario is low energy and has low environmental impact (Austria). The design is also noted as being interesting and the notion of living in a cooler area in nature appears to be appealing (China). Concerns are voiced whether this is an appropriate climate change measure, seeing as it occupies a lot of land per person (Switzerland).

Regarding the possible drivers for the implementation, there appears to be broad agreement on the fact that the increase in heatwaves and heat islands in the course of climate change is a challenge which needs to be addressed, explicitly being named by seven respondents. The need to protect vulnerable people, or people in general, is pointed out by three respondents from Austria and Serbia, which shows that this scenario is recognised as a measure to mitigate risks. Concerns are voiced by a participant from Switzerland that there is a conflict of interest, seeing as space would be occupied which would otherwise serve the public as means for relaxation and leisure. A participant from Italy points out that people would be unhappy to leave their homes. Indeed, while the scenario is currently conceived as a voluntary project, meant to develop solutions for the future, it should not be overlooked that if the climatic situation intensifies and becomes deadlier, this is a measure which could develop to be mandatory. A participant from Australia mentions a few aspects they deemed to be particularly positive, pointing out that this design has low consumption, that it is climate sensitive and that there are no issues with privacy or safety. A participant from Albania identifies private investors and economical drivers as being important possible drivers for implementation, although this is not specified further. Relating to the implementation in their own countries, many respondents appear to identify with the problem of heatwaves (e.g. Italy and Portugal). A respondent from South Africa found this scenario to be applicable in the South African local context. Due to it being so adaptable, even the respondent from Estonia could see it working in their northern context. A point raised by a respondent from China is that ‘this scenario only works in areas which have suitable natural areas (like forests, lakes, parks, trees, streams) in the vicinity’ (Table 12).

**Table 12:** What do you think are barriers and challenges for the implementation in your local framework? (n = 18)

Barrier	Nr. of times chosen	Percentage
Legal	1	6%
Social	6	33%
Political	2	11%
Space constraints	7	39%
Economical	3	17%
Environmental	2	11%
Other	4	22%

Regarding barriers and challenges for the implementation, the aspect ‘space constraints’ was identified by seven respondents, with the respondents believing it is difficult to find an appropriate space, as space is limited and expensive, and it is not easy to find free green areas with good accessibility (Switzerland, Austria). It is also noted critically that occupying space, which is otherwise made available to the public, is a conflict in interest (Serbia, Switzerland).

The aspect ‘social’ was identified by six respondents as being an important barrier for implementation, echoing the concerns already mentioned before, of spaces which are otherwise available to the public, now being used to accommodate a few (Switzerland, Serbia). There seemed to be much hesitation regarding the use of green open spaces for the construction of temporary housing units, as this could create conflicts with the local population, who would feel deprived of such spaces which only serve an advantage to a few users. While this could be justified if the health and safety of the user group is in jeopardy, it is also an option to search for alternatives. For instance, turf could be rolled out at parking lots to create green spaces.

A participant from Italy voiced that he does not believe the people would like this scenario, because they are already used to heatwaves and they would not leave their houses. A respondent from Greece also had concerns, whether people would be willing to leave their homes, stating that it may be possible with the cooperation with professionals working with the elderly, echoing considerations made within the project.

Three participants chose the ‘economical’ aspect as an important barrier, with one elaborating that there is simply no demand (Switzerland). This may be related to this scenario being a pre-emptive measure.

Two respondents chose the ‘environmental’ aspect but did not elaborate further. One respondent who chose ‘other’, however, did bring an environmental argument, stating that open spaces are key resources for handling heatwaves, meaning they should stay unbuilt (Switzerland).

Two respondents chose the ‘political’ aspect, elaborating that there is limited space, and the choice must be made regarding who can be hosted (Kosovo). This could become a critical question, especially if heatwaves become more dangerous to the health of residents.

One participant chose ‘legal’ as an area with important barriers and challenges but did not elaborate further on this. One respondent, who answered with ‘other’, however, brought forth the argument that it will be difficult to obtain a permit to build in a natural area not far from the city, as these areas are usually strictly protected (China). Among the responses in the category ‘other’, a challenge is identified in needing to quickly accommodate a lot of people (Switzerland), the climate type is noted as a big problem, with Nordic countries possibly not struggling with heatwaves to the same extent as more southern countries (Estonia) (Table 13).

**Table 13:** The present scenario was developed for the context of the city of Vienna. Can you imagine this scenario in another city? (n = 18)

Answer	Nr. of times chosen	Percentage
Yes	10	56%
No	4	22%
Not sure	4	22%

Regarding the possibility to develop this scenario in a context other than the city of Vienna, 10 respondents answered with ‘yes’, four responded with ‘no’ and four answered with ‘not sure’, which is substantially lower than for the other scenarios. Cities explicitly named as implementation of this scenario being feasible are as follows: Lausanne, Geneva, Zurich, Paris, Novi Sad, Lille, Brussels, Budapest and Oslo. Suitable areas are identified as southern European cities, Mediterranean climate areas, southern countries and Central Europe, regional areas in Australia affected by fire, flooding, heatwave and air quality changes and coastal areas in New South Wales. Two respondents from Switzerland and Greece could see this scenario being applied across the globe. One respondent from Switzerland, however, did not see this scenario as being particularly relevant to their home country, as heat is not such a big issue in Switzerland. A respondent from China also saw the implementation in their home country critically, stating that this scenario is well-suited for moderate climate, but not for very hot cities with high humidity.

### Scenario Preferences

In the seventh and last part of the questionnaire, the participants were asked which was their favourite scenario among the six scenarios defined within the WWTF-funded project. It was possible to select multiple answers and to elaborate the choices. More than half of the participants chose ‘Life Sharing to Go’ as a favourite scenario, followed by the ‘Shop-hopping box’ and then the ‘GapModule’. A total of three respondents chose ‘Beat the Heat’ and ‘DonAutonom’ respectively, and only one respondent chose ‘Life on Track(s)’ as a favourite scenario (Table 14).

**Table 14:** Which one was your favourite scenario? (n = 18)

**#**	Scenario	Nr. of respondents	Percentage
1	GapModule	4	22%
2	Life Sharing to Go	10	56%
3	Beat the Heat	3	17%
4	Life on Track(s)	1	6%
5	Shop-hopping box	5	28%
6	DonAutonom	3	17%

The use of vacant or unused industrial buildings for communal living appears to have been very interesting to the participants, being deemed as positive in both ‘Life Sharing to Go’ and ‘Shop-hopping box’. When regarding the most popular scenario, ‘Life Sharing to Go’, affordability was also mentioned as a strong suit, with one participant deeming it the most inclusive scenario, as well as the one which would be easiest to implement. One participant noted that there are no conflicting uses, which must be considered as a positive factor. Other positive factors which were brought up were the central location, the visibility raising awareness of the ‘different (and sometimes unequal) temporalities each of us can occupy in the city’, and the ease with which the structure can be mounted and dismounted.

A total of four people chose the ‘GapModule’ as their favourite scenario, finding it to be a project which can easily and quickly be implemented, which is applicable to the local context, has a high acceptability, and which resembles already existing projects (in this case the Pop-Up dorms in Vienna, Austria). Great appeal was found in the use of abandoned areas to increase value and maintain the infrastructure. It was pointed out that due to a lack of funds, abandoned buildings tend not to be maintained or redeveloped, but by reusing them for short periods, this could be an economic solution to the problem. The social aspect also appears to have appealed to many participants, often in the combination with the attributes of there being much space and that it can be used for many purposes. It is noted that this is a solution for young people who make the conscious choice to live this way. It is seen as a way to solve the shortage of affordable housing.

A total of three people chose ‘Beat the Heat’ as their favourite scenario, whereby one participant chose every other scenario, except for Beat the Heat, with the argument that it is the least practical and useful. Another participant, however, argues that it is driven by a real need and is a well-suited scenario for temporary contexts. The affordability is also noted as a positive aspect of the scenario, making it an inclusive project. One participant, who is an urban climatologist in Australia, emphasises that the scenario can contribute to saving many lives in vulnerable communities, calling it ‘a great solution for climate adaptability’. The fact that ‘Beat the Heat’ scored very low overall could be related to the nature of ‘Beat the Heat’ as more of a pre-emptive risk management endeavour, which contains uncomfortable notions of vulnerable individuals, such as the elderly, being pressured out of their homes through outside forces (in this case climate), making it hard to envision. It is also much more difficult to imagine possible future scenarios for something which has not taken place in recent history, such as heatwaves climbing to such extremes, that these types of measures become relevant countermeasures. The scenarios ‘GapModule’ and ‘Life Sharing to Go’, however, contain user groups and situations with greater familiarity, such as people affected by homelessness and refugees with positive asylum status. As for the scenarios not yet in the modelling phase, ‘Shop-Hopping Box’ was regarded particularly positively, due to the reuse of vacant spaces. The scenario was praised for there being no conflicting uses, many retail spaces being vacant, its affordability, the central location and visibility, easy mounting/dismounting operations and no need to occupy other spaces. A total of three respondents chose ‘DonAutonom’ as their favourite scenario, noting that the reuse of old ships is a good idea for a more circular world. One person chose ‘Life on Track(s)’ as their favourite scenario but did not elaborate this further. This was, however, the same individual who chose each scenario, except Beat the Heat. This scenario therefore did not appear to hold much appeal for any of the participants.

When asked if they would live in one of these pop-up environments, 13 of the participants answered ‘yes’, four answered ‘no’ and one abstained. The elaborations of the positive answers showed a strong interest in temporary housing and in communal life. The motivations vary greatly, with some participants stating that they would only do so if the situation required (for instance due to a heatwave or a disaster event), or that they would consider these options in order to save money, or as a good affordable alternative for their lifestyle which involves a lot of moving. A surprising number, however, appear to be interested in temporary housing not out of need, but out of a curiosity of how the experience compares to more traditional housing situations and what benefits can be found in respect to social interactions, freedoms, living climate or reducing environmental impacts. The idea of sharing living space appealed to some of the participants, who believe they would enjoy the experience. Others, on the other hand, viewed the scenarios with sharing concepts as having issues with privacy or safety, and would not voluntarily live in such an environment. Some note these scenarios as perhaps being more suited for younger people than for families who want to settle and have their own private space, with one participant stating: ‘but this might also shift in the future’.

The participants were asked about what keyword they took with them from the session, with the most chosen keyword being ‘adaptability’, followed by the related term ‘modularity’. Another related term which was mentioned is ‘flexible’. Another thematic block seems to surround the topic of environmental sustainability, consisting of the terms ‘circular city concepts’, ‘circular economy’, ‘social and sustainable housing’, ‘reuse’ (chosen twice) and ‘sustainability’. The social dimension also appears to have stuck with many participants, with keywords involving ‘social interaction’, ‘inclusive and exclusive landscapes’, ‘affordable’, ‘social and sustainable housing’ and ‘temporary community’. The temporary nature of housing is common in another set of keywords, consisting of ‘pop-up’, ‘pop-up buildings’, ‘pop-up housing’, ‘temporary housing’, ‘temporary pop-up housing’ and ‘temporary community’. Three keywords which do not fit into any of these loose categories were ‘solution’, ‘inspiring’ and ‘category of housing’.

## Discussion

The PUEs conceptualised within the WWTF-funded project explore innovative and sustainable housing solutions that promote the transition of the construction sector from linear to circular models. Aims include making urban reuse strategies more sustainable in social, economic and environmental terms, in an attempt to find adaptable and flexible paradigms for different urban contexts. The questionnaire sessions allowed interesting insights regarding the possible drivers and barriers of each scenario, highlighting their weaknesses and strengths, focusing on their applicability in the local contexts of the questionnaire respondents.

Thirteen of the 18 (72%) invited respondents participated in the sessions, despite having no direct work experience with temporary housing. Although the conceptualisation of temporary housing as innovative spaces for sustainability is a relatively new topic in literature, it can be seen that it captures the curiosity of the scientific community and that there is interest on the part of experts and professionals to explore the new possibilities that this concept opens up for the construction sector. This finding can be directly linked to one of the last questions, relating to the respondents’ desire to live in one of the pop-up scenarios presented. Fourteen of 18 respondents (76%) said they were open to this possibility, demonstrating a remarkable openness to temporary housing, even if for many this would be a completely new experience. This data is in contrast with some studies on the perception of temporary housing, which is sometimes presented as a type of dwelling in which adequate standards of sustainability in the social, economic or environmental dimension are not met [32, 33].

An interesting finding concerns the position of the participants regarding the requirements that the PUEs should have to achieve sustainability from a social, economic and environmental point of view. It can be noted that all the requirements considered within the project have obtained very high average ratings and therefore they can be considered as being confirmed by the respondents as fundamental points for the realisation of sustainable PUEs. This finding is in line with the sources found in the literature [22, 26–29, 32, 34–40] and, in particular, underlines the importance of two aspects: the deconstructability and reusability of the units and the recyclability of its components at the end of their life cycles. These can be seen as true beneficial attributes of the PUEs as conceptualised here; the possibility of deconstructing the building for future reuse or recycling offers enormous possibilities to enable circularity in the construction sector and contribute to the creation of sustainable cities of the future.

Regarding the important factors for the planning of the PUEs, the respondents confirmed some findings reported in other studies. The answers confirm that the factors behind the planning of temporary strategies can be of the most diverse types and are strongly conditioned by local socio-political conditions, although they share a common interest in economic affordability and environmental sustainability [22, 27–29, 36, 38, 41–47]. This is also underlined by the very high consideration of the respondents regarding the ‘environmental impact’ factor of the PUEs on the places where they are placed. This is of course a reflection of the fact that several of the respondents were recruited at a conference surrounding circular economy, but in general, it can be observed that safeguarding of the environment is now a central theme in every area of the world economy, and it is not surprising that this factor is now seen as essential in the planning phase of any building, permanent or temporary.

On the other side, a result that appears to contrast with the findings in the literature concerns the possibility for the occupants to make adaptations to the unit according to their personal needs and tastes: in the context of the questionnaire sessions, this does not seem to be considered as a particularly important factor. This result was to be expected, as target groups for these questionnaire sessions were planners, architects and engineers with an interest in circular economy, who may not focus so strongly on the social aspects of PUEs. The workshops conducted within the WWTF-funded project involved stakeholders from social fields who strongly emphasised the need for users to be able to claim ownership of the space through means such as adapting it. In light of the fact that it can unfortunately occur that a temporary pop-up environment becomes permanent, even if it was initially intended to have a limited duration, PUEs that are conceived without the requirements of durability and long-term comfort end up being a source of discontent among its users, who sometimes abandon or completely modify these ‘no longer temporary’ houses [27]. Within this paper, it can be observed that the ideas of what temporary housing should be, and where priorities are placed, appear to be quite similar among international experts.

Regarding the Viennese scenarios, the respondents showed relatively high similarities in what they considered positive in the scenarios, but always showed strong differences in the barriers and challenges they identify for the implementation in their framework. This is indicative of it being extremely difficult, or more likely even impossible, to create designs which are universally applicable. The designs will always need an adaptation process if they are to be transferred into another context, the success of which relies heavily on the cooperation of various stakeholders. While many of the respondents could envision the scenarios in another city, the elaborations show that many see the realm of possibilities restricted to cities with similar conditions regarding, for example, culture or climate. This is unsurprising given the fact that the scenarios were very embedded in the framework conditions for the city of Vienna, addressing local needs and opportunities. It is, however, an exacerbating factor that the innovations for sustainability introduced in these temporary housing scenarios are not purely technical in nature, but rather draw on the resources and systems that are already given. It is easier to apply a technical innovation, such as an air conditioning system, in another context, than it is a system drawing on passive cooling technologies, such as is the case for scenario #3 ‘Beat the Heat’. Both require certain preconditions, but while an energy source can be organised with relative ease, natural climatic conditions of an area cannot. Sustainable housing solutions, which rely so heavily on reuse of materials and structures and renewable resources pose a particular challenge for international transferability. Another important factor are the users. At multiple stages of the questionnaire session, the respondents pointed out that a certain user group is not relevant for their country, or that the acceptability of certain ways of living would differ due to cultural reasons. While it is not far-fetched to assume that many scenarios could be adapted to suit other user groups than the ones they were initially intended for, this is a process which must not be underestimated and may prove to be more complex than anticipated. If we regard scenario #2 ‘Life Sharing to Go’, for instance, the whole design relies on a certain willingness of the residents to interact with each other in a particular way, because the layout is conceived this way (e.g. centrally located community kitchens).

## Conclusions

The data obtained from the questionnaire sessions provided useful feedback on the perception of temporary and pop-up environments from international experts from the perspective of circular economy. The responses of the participants were generally positive, showing interest in the potential of PUEs, as a way to contribute to increased sustainability in the construction sector. The responses revealed a number of relevant drivers and barriers for the applicability of the Viennese scenarios to other cities around the globe, spanning from climatic considerations to political and cultural particularities. It was possible to observe that broadly speaking the topics addressed by the Viennese scenarios, e.g. migration and refugees, homelessness and precarious living, affordability of urban housing, climate change adaptations, sustainable building and the like, are global issues, which merely differ in extent and expression, leading to strong resonance with the participants. The scenarios appear to be considered adaptable and flexible enough to be applied in numerous locations, requiring some fine-tuning for the specific local contexts, and being particularly well-suited for the European continent. Heavy design adaptations could be needed if the scenarios are transferred into contexts with significant differences in local cultural and climatic conditions, which may include differences in the type of user groups for which the PUEs are intended. The idea of temporary housing functioning as spaces for more sustainable forms of building and living was received positively by the participants as an intriguing and worthwhile, but also challenging endeavour, requiring intense transdisciplinary cooperation and political will. The present research paper aims to start a broader discussion about the implementation of the Viennese scenarios in an international context. Future steps would include the collection of feedback from a greater number of experts, especially from regions outside of Europe, and consequently a quantifiable approach. This would allow to refine the methodology and evaluate drivers and barriers with comparable parameters. The content of this publication was part of a wider transferability study, where different results are summarised and clustered into methodological and technical packages. The results for the transferability received and analysed so far including the ones from two regional workshops, seem promising. The project team is developing exploitation pathways for different results. Based on a value propositions assessment including needs and benefits, the team plans to apply these concepts in different contexts. In particular, the integration of different disciplines made this research work an interesting experience for all participants. This holistic approach should be maintained in further research works or implementation scenarios.
